# The "Stuck Method" Simplified

**Published:** 1876-05

**Authors:** John D. Wingate


### ARTICLE X

### *The "Stuck Method" Simplified.*

BY JOHN D. WINGATE, D. D. S.

Many who purchased the "Right" to use the Stuck Method, have abandoned it, on account of its complications and uncertainties. Few only can run metal casts into plaster impressions, that are true to the impression. With a few modifications, the subjoined method is shorter than the old one, and will give better satisfaction.

Commencing at the plaster cast, properly trimmed to adjust the plate to the soft and hard parts of the palate, cover the cast with the thinnest of Stuck's (pure tin) polisher plate. Adjust by rubbing down with the thumbs; finish up by burnishing lightly, avoiding folds or wrinkles as much as possible. The edges of the plate should extend beyond the limits of waxing up, so as to be able to hold it on the cast, while removing the base plate, and the case when waved up.

Tea lead, say three thicknesses, manipulated successively on the cast, and fastened together with a little wax, although quite heavy, will answer for a base plate, but gutta percha is much better. The tin on the cast should be covered with pulverized soap stone, to prevent the gutta-percha from sticking. After cooling and trimming, get the bite as usual, grind on the teeth and wax up, finishing the lingual surface as carefully as it is desired to have the finished plate. After setting the case into the bottom part of the flask oil the lingual surface slightly, run a plaster cast over the base plate only, and even with the cutting edges of the teeth. This cast after hardening, is taken out and polished. Over this manipulate polisher plate No. 2 (heavier than the first.) Polish with a cloth moistened with benzine before replacing on the cast, as this is to make the polished lingual surface with the plate. Burnish the projecting edges of tin egainst the teeth and back edge of the plate. The upper portion of the flask may now be filled with plaster, as usual. After opening the flask, and removing the base plate and wax, it is well to again wash with benzine, as on the cleanliness of the tin plates depends the beauty and fine finish of the rubber plate. The polisher plate on the lingual surface should be pretty heavy as it leaves a better surface. It is always best to measure the rubber before packing, to prevent undue pressure, which may have injurious effects, even on the cast.—*Dental Advertiser.*

